# Risk of obesity in immigrants compared with Swedes in two deprived neighbourhoods

**DOI:** 10.1186/1471-2458-9-304

**Published:** 2009-08-22

**Authors:** Johan Faskunger, Ulf Eriksson, Sven-Erik Johansson, Kristina Sundquist, Jan Sundquist

**Affiliations:** 1 Department of Neurobiology, Health Care Sciences and Society/Center for Family and Community Medicine, Karolinska Institutet, Stockholm, Sweden; 2 Deparment of Clinical Science, Center for Primary Health Care Research, Lund University, Malmö, Sweden; 3 Stanford Prevention Research Center, Stanford University, Palo Alto, Califorina, USA

## Abstract

**Background:**

Despite a strong social gradient in the prevalence of obesity, there is little scientific understanding of obesity in people settled in deprived neighbourhoods. Few studies are actually based on objectively measured data using random sampling of residents in deprived neighbourhoods. In addition, most studies use a crude measure, the body mass index, to estimate obesity. This is of concern because it may cause inaccurate estimations of the true prevalence and give the wrong picture of the factors associated with obesity. The aim of this study was to estimate the prevalence of, and analyse the sociodemographic factors associated with, three indices of obesity in different ethnic groups settled in two deprived neighbourhoods in Sweden.

**Methods:**

Height and weight, waist circumference and body fat percentage were objectively measured in a random sample (n = 289). Sociodemographic data were obtained through a survey. Established cut-offs were used to determine obesity. Country of birth was categorized as Swedish, Other European, and Middle Eastern. Odds ratios were estimated by unconditional logistic regression.

**Results:**

One third of the sample was classified as obese overall, with 39.0% of women being abdominally obese. After adjusting for age, we found higher odds of obesity in Middle Eastern women than in Swedish women regardless of outcome with odds ratios ranging between 2.74 and 5.53. Men of other European origin had higher odds of BMI obesity than Swedish men. Most associations between country of birth and obesity remained in the full model.

**Conclusion:**

This study demonstrates the magnitude of the obesity problem and the need for prevention programmes targeting native and immigrant adults in deprived neighbourhoods in Sweden. The initiatives should also focus on particular groups, e.g. immigrant women and those experiencing economic difficulties. Further studies are needed on behavioural and environmental factors influencing the risk of obesity in residents settled in deprived neighbourhoods.

## Background

Obesity is rapidly increasing worldwide and contributes to chronic diseases and increased risks of morbidity, disability and mortality [1,2] It is estimated that 30–80% of adults in the European region are overweight or obese, with approximately 150 million individuals being obese [1] and resulting in 1 million deaths and 12 million life-years of ill-health annually [3]. Likewise, several studies show that the prevalence of obesity has increased dramatically in Sweden [4-8]. For example, Swedish national data based on self-reported height and weight (body mass index, BMI) indicate that BMI obesity doubled from 5 to 10% between 1980 and 2005 [8]. Another national study concluded that the prevalence of BMI obesity increased from 8.8 to 11.9% in women and from 6.6 to 10% in men between 1980 and 1997 [5]. There is a strong social gradient in the prevalence of obesity with higher rates among people with low-level education, low socioeconomic status and in many immigrant groups. Those with a low level of education have about double the prevalence of BMI obesity of those with a high level of education, among both men and women [8]. Higher rates of BMI obesity have been found among adolescents from low-level socioeconomic families than among adolescents from higher-level socioeconomic families [9]. Obesity may also adversely affect socioeconomic status and even decrease the likelihood of being employed [10]. In addition, the role of country of birth and immigration in BMI obesity has been investigated. For example, immigrant men and women from Turkey settled in Sweden had an obesity prevalence of 10 and 16%, respectively, in the late 1990s [11]. Finnish men and women settled in Sweden had a BMI obesity prevalence of 17 and 19%, respectively, in 2001 [8]. Another important issue concerns deprived neighbourhoods considering that a Swedish study using hierarchical analyses indicated that the prevalence of BMI obesity increased with increasing levels of neighbourhood deprivation. [12] An American study has recently reported nearly double the prevalence of BMI obesity in deprived neighbourhoods compared to middle/high income neighbourhoods (23 vs 14%) [13].

Despite a strong social gradient in the prevalence rates, few studies have targeted deprived neighbourhoods to investigate obesity and the correlates thereof. It is important to investigate deprived neighbourhoods because disability, chronic disease and premature death rates are higher in these neighbourhoods than in more affluent ones and obesity is a major risk factor for these outcomes [1]. However, the majority of studies are based on self-reported height and weight [1], which is of concern because self-reported data tend to underestimate the actual prevalence of obesity [14-16]. A review recently concluded that the difference between reported and objectively measured BMI obesity prevalence in scientific studies ranges from 0.0% to 49.6% of the actual prevalence [16]. In a Swedish study, the underestimation of the prevalence of BMI obesity based on self-reports was 8% [17]. Furthermore, socioeconomic differences in the validity of self-report instruments for estimating BMI have been reported in Sweden [18]. In addition, the BMI has some well-established limitations with regard to estimating obesity, e.g. it does not discriminate between fat and fat-free mass [19]. Several papers have therefore called for studies based on more accurate and direct measures of fatness and obesity [20,21].

The novelty of this study is the use of three objectively measured indices of obesity – BMI, waist circumference (WC) and body fat percentage (BF%) – in men and women from different ethnic (country of birth) groups settled in two deprived neighbourhoods in order to examine the prevalence of obesity and the sociodemographic factors associated therewith. The use of three different measures can yield more accurate estimates of the actual prevalence of obesity and may provide newer insights into the sociodemographic variables that influence the risk of obesity than when relying on BMI alone. In addition, little prior research has investigated whether the correlates of obesity differ between men and women [22]. This study is important for increasing our understanding of (a) the magnitude of the obesity problem in deprived neighbourhoods, (b) the sociodemographic factors linked to obesity and (c) salient groups to target in preventive strategies.

The aims of the study are, firstly, to estimate the prevalence of obesity in immigrants from the Middle East, other Europeans and in Swedes, using objective measures of BMI, WC and BF% and, secondly, to analyse the association between country of birth and obesity after adjusting for the following variables: age, gender, economic difficulties, education and duration of residence in the neighbourhood.

## Methods

### Design and subjects

This study is part of an ongoing research project evaluating the influence of comprehensive neighbourhood renewal on physical activity and health and social outcomes in children and adults from a very deprived neighbourhood in Södertälje, which is a town situated south of Stockholm in Sweden. Anthropometric measures and sociodemographic variables used in this study constituted part of the evaluation.

Simple random samples of residents aged 18–65 (n = 1400) were obtained from two deprived neighbourhoods south of Stockholm through the local Government. A total of 3168 adults between 18 and 65 years of age lived in the neighbourhood Hovsjö and 2898 adults aged 18–65 in the neighbourhood Geneta.

There were three exclusion criteria in the study:

1. No listed, or an incorrect, phone number (land line or mobile; n = 666)

2. Disabled or severely ill subjects (n = 8)

3. Subjects who did not reside in the designated neighbourhoods (n = 48) (overcoverage).

A total of 722 subjects were excluded from the study, resulting in an eligible sample of 678 individuals. A letter in Swedish and Arabic containing information about the study was posted to the remaining sample. Repeated attempts over the course of one month were made to contact subjects by telephone. Subjects agreeing to participate (n = 317; response rate = 47% of the eligible sample) were sent a confirmation letter containing additional information, e.g. to wear appropriate clothing for the relevant tests. Their written informed consent was obtained. Eleven individuals from other countries outside Europe and the Middle East were not included in the present analysis and 17 were excluded due to incomplete data; thus the present study was based on a sample of 289 individuals.

Local Government statistics showed that a high percentage of the residents had an immigrant background, primarily from the Middle East, including Turkey, and from European countries, mainly Finland. The proportion of adult residents born outside Sweden was approximately 67% in the two neighbourhoods according to statistics from the local government (in 2006).

### Care Need Index – a measure of deprivation

The level of deprivation was estimated using the Care Need Index (CNI). The CNI was originally developed for research purposes and is now being used in several Swedish counties for the distribution of primary health care resources. It is based on the proportion of the following sociodemographic factors in the neighbourhood: elderly persons living alone, children under age 5, unemployed people, low educational status, single parents, high mobility and foreign-born people from Southern and Eastern Europe, Asia, Africa and South America. CNI is correlated with other composite instruments assessing neighbourhood deprivation such as the Townsend score [23,24]. The scale is constructed so that the higher the CNI score, the more deprived the neighbourhood. In the early 1990s the CNI was calculated for all neighbourhoods in Sweden – a total of 8509 neighbourhoods. The CNI scores ranged from -76.4 (most affluent neighbourhood) to 53.5 (most deprived neighbourhood) [25]. The calculation of the CNI in the present study was based on data from 2006, but the range varies only slightly between years. The CNI was 50 in the neighbourhood Geneta and 52 in the neighbourhood Hovsjö, indicating that the two neighbourhoods were very deprived.

### Non-response analysis

Subjects declining participation were prompted to answer a set of questions pertaining to age, gender, country of birth and level of education in order to allow comparisons with participating subjects. A total of 228 subjects agreed to answer these questions while 134 either declined or could not be reached. Compared to participants, non-participants were slightly younger (age 41.2 ± 14.8 vs 43.9 ± 13.6) and were more likely to be of Swedish origin (58% among non-participants vs 31% among participants). Fewer non-participants (14%) than participants (23%) reported a higher level of education. However, non-participants were less likely to report no or low-level education compared to participants (28% vs 38%). In addition, a higher percentage of men was found among non-participants (56%) than among participants (51%).

### Anthropometric measurements

Questionnaire and anthropometric data were collected during March-June 2008 in central locations in the respective neighbourhoods. Staff were trained to conduct the assessments based on a set standard of procedures. Standardized anthropometric measurements were performed on subjects dressed in light indoor clothing without socks and shoes. Height was measured to the nearest 0.1 cm using a portable stadiometer (Seca 67029). BMI (kg/m^2^) provides information about body volume (area) and has become the most common measure identifying obesity in social science research. In epidemiological studies, there is a strong association between BMI and the risk of medical complications and mortality [26,27]. WC was measured to the nearest 0.1 cm using a tape measure on standing subjects at the end of gentle expiration. WC was obtained midway between the lower border of the rib cage and the superior border of the iliac crest. The tape measure was placed around the bare midriff of each participant. WC provides an estimate of body girth at the level of the abdomen and is an indicator of central adiposity and body shape. A high WC, out of proportion to total body fat, is an independent predictor of risk factors and morbidity [28]. BMI and WC are generally highly correlated [29], but there is evidence that WC is better at identifying individuals at cardiometabolic risk (e.g. elevated blood pressure, dyslipidaemia, type 2 diabetes, hyperglycaemia) than when relying on BMI alone. This is particularly true of type 2 diabetes where WC is a stronger predictor than BMI [30,31]. This scenario has also been established in immigrant populations, e.g. in Bosnian refugees in Sweden and Mexican Americans in the United States [32,33]. WC scores were categorized according to established guidelines for adults [28].

There are several methods for estimating BF%. In this study, it was measured by bioelectric impedance analysis (BIA), which provides information about the proportion and distribution of body fat as well as lean body mass. BIA has been validated in obese patients [34] and shows good correlation with established golden standard procedures for identifying and measuring body fatness such as dual energy X-ray absorptiometry [35,36]. BIA with an integrated digital scale was used to assess body fat mass and body weight (Tanita, model BC 418). The assessment followed the instructions recommended by the manufacturer [37]. The recorded weight of the subjects was reduced by 1 kilogram to compensate for light clothing. Body fatness scores were categorized according to specific established guidelines for age and gender.

### Outcome variables

All outcome variables were measured objectively.

*Obesity *was defined as BMI ≥ 30 and was calculated as body weight (kg) divided by height (m) squared (kg/m^2^).

*Abdominal obesity by waist circumference (WC) *was defined as ≥ 102 cm for males and ≥ 88 cm for females.

*Obesity by body fat percentage (BF%) *(defined by gender and age group) for men age 18–39 ≥ 25%, age 40–59 ≥ 28%, age 60–65 ≥ 30% and for women age 20–39 ≥ 39%, age 40–59 ≥ 40, age 60–65 ≥ 42%.

### Sociodemographic data

Socioeconomic data and demographics were obtained from a self-administered questionnaire:

*Gender*: male or female.

*Age *was included as a continuous variable (linearly related to the outcomes) and centred around its mean (44 years).

*Country of birth *was categorized as Swedish, other European (of which half the sample was born in Finland) and Middle Eastern (including Turkey).

*Educational status *was classified according to three categories: Low-level education (no education or compulsory education only), Intermediate-level education (secondary school), and High-level education (college/graduate education). In the weighted logistic regression model, the categories for Intermediate and High-level education have been combined.

*Economic difficulties during the past year *comprised three categories: None, once and several times. Table 1 displays the proportion of residents reporting economic difficulties once or several times during the past year.

**Table 1:** Characteristics (means and standard deviations, percentages) of the study population by country of birth.

**Variable**	**Swedish** **(n = 90)**	**Other European** **(n = 33)**	**Middle-Eastern** **(n = 166)**	**P value**
Age (years)	44.8 ± 16.1	46.1 ± 14.2	43.7 ± 11.9	0.50 (b)
Gender (%)				
Male	44 (n = 40)	58 (n = 19)	54 (n = 89)	0.27 (c)
Female	56 (n = 50)	42 (n = 14)	46 (n = 77)	
Duration of residence in neighbourhood (years)	13.8 ± 11.1	11.9 ± 10.8	10.9 ± 9.4	0.23 (b)
Duration of residence in Sweden (years)	-	26.6 ± 13.7	16.0 ± 11.2	0.0001 (b)
Economic difficulties during the past year (a) (%)	22.2	21.2	42.8	0.01 (b)
Education (%)				
Low	30.0	39.4	41.0	0.053 (c)
Intermediate	48.9	48.5	32.5	
High	21.1	12.1	26.5	

(a) Having economic difficulties once or more often during the past year. (b) Kruskal- Wallis (c) Chi-square.

*Duration of residence in the neighbourhood *was divided into three groups: < 5, 5–9, and ≥ 10 years.

*Duration of residence in Sweden (years) *is shown in Table 1 (descriptive data) and is not included in the models.

### Statistical analyses

Continuous variables are presented as the mean ± the standard deviation (SD), whereas the distributions of the categorical variables are presented as percentages (Tables 1 and 2).

Differences in distribution were tested by the chi-square test and differences betweens means by the Kruskal-Wallis test. The association between sociodemographic factors and the odds of being obese was estimated by weighted logistic regression analysis. Post-stratified weights [sex*age (18–24, 25–34, 35–44, 45–54 and 55–65)] were calculated as the inverse inclusion probabilities of the respondents [N_i_/n_is_], where N_i _is the size of the population in stratum i and n_is _is the number of respondents in stratum i. The weights ranged between 12 and 42 to account for different response rates in subgroups. The results are shown as odds ratios (ORs) with 95% confidence intervals (CIs).

The statistical package STATA 10 (StataCorp LP, Texas, USA) was used in all analyses.

Ethical approval of the study was granted by the central ethical review board, the Swedish Research Council (Dnr 2007/196-31 and Dnr Ö37-2006).

## Results

Characteristics of the study population are shown in Table 1. There were no statistically significant differences between the groups studied except that immigrants from the Middle East reported about two times higher percentages of economic difficulties (p = 0.01) than Swedes. The average age was 44 with the Middle East group being the youngest and the Other European group the oldest. The duration of residence in the neighbourhood ranged between 14.5 years (Swedes) and 11.4 years (immigrants from the Middle East). There was a significant difference in duration of residence in Sweden between the two foreign-born groups.

The prevalence of obesity defined as BMI, WC and BF% and by country of birth and gender is shown in Table 2. The prevalence of obesity was high in all groups studied with approximately one third of the sample being obese. Particularly Middle Eastern women had a very high prevalence of obesity.

**Table 2:** Prevalence (%) of obesity by body mass index (BMI), waist circumference (WC) and body fat percentage (BF%) in the study population by country of birth and gender.

**Outcome**	**Total sample**		**Swedish**		**Other European**		**Middle East**	
	Men	Women	Men	Women	Men	Women	Men	Women
	
**BMI:** **Obese (BMI ≥ 30)**	28.4	31.2	17.5	16.0	31.6	21.4	32.6	42.9
**WC^**1)**^:** **Abdominal obesity**	25.7	39.0	25.0	30.0	26.3	50.0	25.8	42.9
**BF%^**2)**^:** **Obese**	27.0	30.5	22.5	24.0	21.1	14.3	30.3	37.7

1) Men and women were defined as abdominally obese if their waist circumference (WC) was ≥ 102 cm (men) or ≥ 88 cm (women).

2) Body fat percentage. The definition of obesity varied by age and gender: Men: age 18–39 ≥ 25%, age 40–59 ≥ 28%, age 60–65 ≥ 30%. Women: age 20–39 ≥ 39%, age 40–59 ≥ 40%, age 60–65 ≥ 42%.

Age-adjusted odds ratios by gender are shown in Table 3, comparing the immigrant groups with Swedish-born individuals (Reference). Among men, immigrants of Other European origin had significantly higher odds of obesity than the Swedish group (OR = 1) only in terms of BMI (OR = 4.14, CI = 1.10–15.5). Women from the Middle East had higher odds of obesity than Swedish women (OR = 1) irrespective of outcome, with odds ratios ranging between 2.74 and 5.53.

**Table 3:** Age-adjusted odds ratios (ORs) with 95% confidence interval (CI) for BMI obese, waist circumference (WC) abdominal obesity and body fat percentage (BF%) obesity by gender, obtained by weighted logistic regression analysis.

**Outcome variable**	**Gender**	**Swedish**	**Other European**	**Middle Eastern**
BMI obese	Men	1 (Reference)	4.14 (1.10–15.5)	2.48 (0.99–6.24)
WC abdominal obesity		1 (Reference)	1.89 (0.43–8.33)	1.16 (0.48–2.83)
BF% obesity		1 (Reference)	1.57 (0.37–6.60)	1.84 (0.77–4.40)
BMI obesity	Women	1 (Reference)	0.91 (0.21–3.83)	5.53 (1.91–16.1)
WC abdominal obesity		1 (Reference)	1.64 (0.50–5.36)	2.74 (1.15–6.56)
BF% obesity		1 (Reference)	0.43 (0.08–2.19)	3.18 (1.36–7.46)

Most results were analysed by gender; however, in Table 4 men and women are analysed together, as no interaction was found between gender and country of birth.

**Table 4:** Odds ratios (ORs) with 95% confidence intervals (CIs) for BMI obesity, waist circumference (WC) abdominal obesity and body fat percentage (BF%) obesity, obtained by weighted logistic regression.

**Sociodemographic variables**	**BMI Obesity**	**WC Abdominal obesity**	**BF% obesity**
Country of origin			
- Swedish	1 (Reference)	1 (Reference)	1 (Reference)
- Other European origin	2.79 (1.06–7.36)	1.96 (0.75–5.10)	1.11 (0.36–3.42)
- Middle Eastern origin	3.10 (1.51–6.40)	1.63 (0.82–3.26)	2.22 (1.15–4.28)
Age (years)			
- continuous and centred	1.03 (1.01–1.06)	1.06 (1.03–1.08)	1.03 (1.00–1.05)
Gender			
- Male	1 (Reference)	1 (Reference)	1 (Reference)
- Female	1.23 (0.70–2.16)	2.15 (1.21–3.81)	1.34 (0.76–2.36)
Economic difficulties in the past year			
- No	1 (Reference)	1 (Reference)	1 (Reference)
- Yes, once	1.32 (0.62–2.78)	1.54 (0.70–3.26)	0.59 (0.25–1.40)
- Yes, several times	2.12 (1.00–4.54)	2.29 (1.07–4.93)	1.82 (0.87–3.81)
Education			
- Low	1 (Reference)	1 (Reference)	1 (Reference)
- Intermediate/high	0.73 (0.38–1.40)	0.81 (0.43–1.52)	1.06 (0.55–2.05)
Duration of residence in neighbourhood (years)			
- 0–4	1 (Reference)	1 (Reference)	1 (Reference)
- 5–9	1.42 (0.66–3.08)	1.58 (0.71–3.51)	2.10 (0.96–4.58)
- 10-	0.74 (0.37–1.47)	0.74 (0.37–1.49)	1.00 (0.49–2.04)

In Table 4 the association between obesity and country of birth is shown with adjustments for age, gender, economic difficulties, education and duration of residence in the neighbourhood. Immigrants from the Middle East had significantly higher odds for both BMI obesity and BF% obesity (OR = 3.10, CI = 1.51–6.40 and OR = 2.22, CI = 1.15–4.28, respectively) than Swedes (OR = 1). Immigrants from Other European countries had significantly higher odds of BMI obesity (OR = 2.79, CI = 1.06–7.36) than Swedes. Women had about double the odds of WC abdominal obesity than men. Those with economic difficulties encountered several times during the past year had about double the odds of obesity, measured by BMI and WC, than those with no economic difficulties. Education was not associated with any of the obesity measures.

## Discussion

The main finding of this study was the high prevalence of obesity in all groups including the reference group, subjects born in Sweden, and regardless of outcome. We found higher age-adjusted odds of obesity in Middle Eastern women than in Swedish women regardless of outcome. Men of other European origin had higher odds of BMI obesity than Swedish men. Most associations between country of birth and obesity remained in the full model.

Although the finding of a high prevalence of BMI obesity in immigrant groups is in line with previous research, e.g. Sundquist et al [8] and Gadd et al [11], it is important to note that the prevalence established in our study was much higher, being up to three times as high. The prevalence of BMI obesity among men and women from the Middle East was, however, higher than in another Swedish study based on a sample from the entire population [11]. A similar pattern was evident among native Swedes with a much higher prevalence of BMI obesity than in a previous population-based study [8]. A Turkish study with a representative national sample [38] classified 21% of men and 40% of women as BMI obese.

A nationally representative study from Greece based on objectively measured WC [39] reported that 26.6% of men and 35.8% of women were WC obese, which is in accord with the prevalence of WC obesity established in our study. An interesting result is that 50% of the women of Other European origin had WC abdominal obesity, while only 14.3% were categorized as BF% obese. Thus, it seems that the use of several objective indices of body fatness can be useful when studying obesity in adults from different ethnic groups as WC and BF% provided different pictures of the prevalence of obesity. However, these results should be interpreted with caution since the Other European group was small, e.g. there were only 14 women.

We found higher odds of obesity in Middle Eastern women regardless of objectively measured outcomes than in Swedish women and higher odds of BMI obesity in men of other European origin than Swedish men. It is important to note that the Swedish reference group also had a very high prevalence of obesity, which influences the odds ratios.

In the analysis of sociodemographic correlates, individuals reporting economic difficulties several times during the past year had higher odds of obesity than those without such difficulties, which is in line with previous research on socioeconomic variables [1]. Surprisingly, educational status was not associated with obesity, which is in contrast to a previous Swedish study [8] and most other recent research in countries in Western Europe [1]. This lack of association may be due to differences in data collection, i.e., self-reported data in previous studies versus objectively measured indices of obesity in this study. The result may also be due to the fact that many immigrants living in deprived neighbourhoods could be highly educated but with few realistic opportunities to reach higher socioeconomic strata.

In terms of objectively measured indices of obesity, few studies have examined specific communities or deprived neighbourhoods. A Spanish study [40] using randomly selected adults estimated the prevalence of BMI obesity to be 23.6% for men and 36.5% for women in the Canarian community of Guía. A recent American study conducted in deprived neighbourhoods classified 45% of the sample as BMI obese [41].

Several studies have found associations between neighbourhood deprivation and obesity [12,42,43]. The mechanisms behind neighbourhood environments and obesity are not well understood. Recent studies have suggested that the associations between neighbourhood disorder and obesity are mediated by psychological stress [44]. Feeling unsafe in neighbourhoods is associated with obesity in young mothers [45]. Lower socioeconomic status has been associated with being less health conscious and having several unhealthy lifestyle habits [46]. Previous studies have demonstrated relationships between the built-up environment and weight-related outcomes [47-49]. However, studies in ethnically diverse neighbourhood environments based on objective measurements are few in number.

The key strength and novel contribution of the present study is the use of three objectively measured indices of obesity. To the best of our knowledge, no previous study has objectively measured obesity in random samples of residents from deprived neighbourhoods, taking sociodemographic correlates into account. This study also has some limitations. For example, the high non-response rate might introduce a selection bias. However, we have, to some extent, taken a possible non-response bias into consideration by including a weighting system based on gender and age in the statistical analysis. In addition, we achieved a representative sample in terms of ethnicity (31% were of Swedish origin compared to 33% in the population register in the study neighbourhoods). Another limitation concerns the possible socioeconomic selection bias caused by the exclusion of residents without a listed correct phone number. This possible selection bias is, however, most probably a minor problem in Sweden, where social inequalities are less pronounced than in many other countries. This means that socioeconomic differences are unlikely to affect access to a telephone. A third limitation is the relatively small sample (n = 33) in the immigrant group Other European origin resulting in wide confidence intervals in Table 3, which suggests that only large effects might be captured.

## Conclusion

This study demonstrates the magnitude of the obesity problem and the need for prevention programmes targeting both native and foreign-born adults in deprived neighbourhoods. These programmes could focus particularly on certain population groups, such as immigrant women and those experiencing economic difficulties.

## Competing interests

The authors declare that they have no competing interests.

## Authors' contributions

All authors conceived and designed the study, were involved in the analyses and drafted the manuscript. JF and UE also collected the data.

## Pre-publication history

The pre-publication history for this paper can be accessed here:


